# Sjogren Disease and Nephrolithiasis: A Case Series and Review of the Literature

**DOI:** 10.3390/clinpract15120225

**Published:** 2025-11-28

**Authors:** Ben Schroeder, Chokkalingam Siva, Chen-Chung Steven Liu

**Affiliations:** 1 Department of Medicine, University of Missouri System, 1 Hospital Dr., Columbia, MO 65212, USA; 2 Division of Rheumatology, Department of Medicine, University of Missouri System, 1 Hospital Dr., Columbia, MO 65212, USA; sivac@health.missouri.edu

**Keywords:** Sjogren disease, nephrolithiasis, renal tubular acidosis, chronic kidney disease, pathophysiology

## Abstract

**Background:** Primary Sjogren Disease (pSD) is a chronic autoimmune disease characterized by a classic triad of keratoconjunctivitis sicca, xerostomia, and polyarthritis. The primary pathological feature of pSD is lymphoplasmacytic infiltration in glandular epithelial tissue, often affecting the salivary and lacrimal glands, leading to classic sicca symptoms (ocular and oral dryness). Sjogren Disease (SD) can be categorized as “primary” when occurring independently or “secondary” when accompanying another autoimmune connective tissue disorder such as rheumatoid arthritis, systemic lupus erythematosus, or systemic sclerosis. Additionally, systemic disease is common in pSD and can manifest with kidney dysfunction resulting in nephrolithiasis and distal renal tubular acidosis (dRTA). **Methods:** This report details a case series drawing patients from the literature as well as patients from our institution which serves to demonstrate key points in clinical hallmarks. We utilize a literature search with key words Sjogren Disease, nephrolithiasis, renal tubular acidosis, and nephrocalcinosis in addition to pSD patients with concomitant nephrolithiasis at our institution to characterize clinical and serologic findings as well as treatment modalities. **Results:** We find well demonstrated clinical hallmarks such as female predominance and presence of dRTA amongst the cohort of pSD patients. We also find that further research on pSD serologies could prove beneficial in risk stratifying those most likely to develop renal disease and nephrolithiasis. Furthermore, we review signs, symptoms, pathophysiology, and management of SD with added emphasis on associated renal disease including nephrolithiasis and dRTA. **Conclusion:** Overall, pSD associated renal disease remains an area of ongoing research and further study on patient serologies may aid clinicians in better serving and surveilling patients at risk of systemic involvement.

## 1. Introduction

Primary Sjogren Disease (pSD) is a chronic inflammatory autoimmune disease characterized by a classic triad of keratoconjunctivitis sicca, xerostomia, and polyarthritis and was first identified by Swedish ophthalmologist Henrik Sjögren (1899–1986) [1]. The primary pathological feature of pSD is lymphoplasmacytic infiltration in glandular epithelial tissue, often affecting the salivary and lacrimal glands, leading to classic sicca symptoms (ocular and oral dryness) [2]. Sjogren Disease (SD) can be categorized as “primary” when occurring independently or “secondary” when accompanying another autoimmune connective tissue disorder such as rheumatoid arthritis, systemic lupus erythematosus, or systemic sclerosis [2,3]. It predominantly affects middle-aged women in their fourth to sixth decades of life, with a female-to-male predominance of 9:1 [3]. Additionally, systemic disease is common in pSD and can frequently manifest with kidney dysfunction resulting in nephrolithiasis.

## 2. Primary Sjogren Disease Case Series and Analysis

In our case series incorporating both pSD patients from clinics at our institution (Schroeder et al.) and from the literature [4,5,6,7], we review thirteen patients with pSD and associated nephrolithiasis and/or nephrocalcinosis. This review of the current literature as well as our case series from patients at our institution displays some common trends in demographics, disease manifestations, and management amongst patients with pSD. The noted serologic findings amongst this cohort are also of interest given the high prevalence of positive autoantibodies.

In order to find relevant cases of pSD with associated renal disease including dRTA and nephrolithiasis, we performed a Medline literature search with keywords Sjogren Disease, nephrolithiasis, renal tubular acidosis, and nephrocalcinosis. Based on this search mechanism, all identifiable relevant cases and their crucial data points have been summarized in Table 1 for review. Upon examination of this patient data, we find notable trends that strongly mirror what is already known about pSD. Among all examined cases of nephrolithiasis in patients with pSD, only two out of the thirteen were male patients. This stratification of two male patients and eleven female patients reflects data on pSD, which often suggests a female-to-male ratio of 9:1. Additionally, nine of the thirteen patients were found to have evidence of dRTA. This predominance of dRTA amongst patients with pSD and nephrolithiasis is also supported by known pathophysiologic mechanisms for which dRTA (potentiated by pSD) induces kidney stone formation. Lastly, we find that many patients reviewed in this case series received treatment with hydroxychloroquine; specifically, ten of the thirteen patients. Hydroxychloroquine was also the only notable therapy exhibiting consistency amongst the patients reviewed. While hydroxychloroquine has not been shown to treat renal disease in pSD, such as dRTA and nephrocalcinosis, hydroxychloroquine is recommended as first-line treatment of classic sicca and arthritis symptoms. Overall, these data points by in large reveal a typical cohort reflective of pSD patients even amongst those exhibiting renal involvement of nephrolithiasis and/or nephrocalcinosis likely precipitated by pSD-mediated dRTA.

The patients’ serologies, meanwhile, are of greater intrigue. Much of the data on serologies in pSD details diagnostic utilization of ANA (antinuclear antibody), SSA (Sjogren Syndrome A antibody), and SSB (Sjogren Syndrome B antibody) titers in addition to other serologic tests such as rheumatoid factor (RF). Positive ANA serology, for example, is present in about 80% of pSD patients [8]. Rates of positive SSA and SSB titers however vary somewhat throughout the literature. Positive SSA titers are frequently reported to be present in 50–70% of pSD patients, and rate of positivity for SSB titers is often in the range of 25–40% [9,10,11]. Additionally, titer positivity has also been found to be associated with an increase in disease severity [11]. These figures help demonstrate that positive serology can vary amongst those diagnosed with pSD and of course that seronegative disease is also possible. We find that, of the thirteen patients with pSD and nephrolithiasis and/or nephrocalcinosis, several patients display positive serology. Of the thirteen patients in our review, eleven had positive ANA titers (84.6%), ten had positive SSA titers (76.9%), and seven had positive SSB titers (53.8%). While little is known about the predictive value of serology for renal disease, it is established that serology can elucidate risk for increased generalized disease severity. Furthermore, recent research has emerged showing that positive rheumatoid factor serology is predictive for RTA in patients with pSD amongst some other factors including chronicity of pSD and thyroid disease [12]. This study also posits a fascinating predictive nomogram for risk-stratifying a pSD patient’s likelihood to develop RTA [12]. Given this new evidence paired with the high rate of positive serology seen in our case review patients, we should recognize that further research is needed to best characterize serologic findings and any association with pSD-related renal disease, should it exist.

## 3. Sjogren Disease Review

Diagnosing pSD typically necessitates at least one sign of ocular or oral dryness alongside at least one immunologic abnormality, such as serum anti-SSA antibodies or focal lymphocytic sialadenitis. Several classification criteria for SD, designed primarily for clinical research studies, have been proposed and are commonly used to aid diagnosis in clinical practice. The American-European Consensus Group’s (ACEG) criteria, established in 2002, are frequently employed, but the American College of Rheumatology (ACR)–European League against Rheumatism (EULAR) criteria, validated in 2016, are the most recent [13,14]. Both sets of diagnostic criteria can be reviewed in Table 2. Additionally, while the presence of positive antinuclear antibody (ANA) titers and rheumatoid factor (RF) titers is common, they are not part of the diagnostic criteria for SD [15].

Clinical signs of SD stem from exocrine gland manifestations as well as extraglandular disease features [3]. Exocrine gland manifestations, characterized by lymphocytic infiltration and epithelial tissue destruction in the lacrimal or salivary glands is the hallmark of the disease, and nearly all patients exhibit a classic sicca syndrome, including ocular or oral dryness commonly manifesting with dysphagia, difficulty speaking for long durations, dental carries, ocular irritation, itching, foreign body sensation, and blurry vision [2,15,16,17,18]. Furthermore, SD can affect nearly any organ system, causing fatigue (reported by 70–80% of patients with pSD), fibromyalgia (present in 15–30%), tubulointerstitial nephritis, autoimmune primary biliary cholangitis, obstructive bronchiolitis, interstitial lung disease, palpable purpura, cryoglobulinemia-associated glomerulonephritis, peripheral neuropathy, and arthritis [15,19,20,21,22,23].

Management of pSD heavily relies on symptom control but can include immunosuppression as well. Clinical practice is heavily guided by recent EULAR recommendations published in 2020. First-line symptom control measures include targeting sicca and joint-related disease. Arthralgia and arthritis can both be managed with over the counter or prescription non-steroidal anti-inflammatory drugs (NSAIDs) [24]. Additionally, artificial saliva and artificial tears are safe and often effective for sicca symptoms [24]. Salivary stimulation with non-pharmacologic gustatory stimulants in the form of lozenges is also recommended [24]. For more moderate sicca disease, pharmacologic stimulation with pilocarpine or cevimeline may also be employed [24]. When it comes to systemic extraglandular disease, however, therapies targeting the immune system are indicated. Symptomatic treatment with glucocorticoids followed by the addition of chronic steroid-sparing therapy with hydroxychloroquine or classic disease-modifying anti-rheumatic drugs (DMARDs) such as methotrexate or leflunomide is recommended [24]. Of note, choice between various DMARDs is often based on patient comorbidities and tolerability as there is insufficient data showing superiority of any specific agent [24,25]. These systemic therapies may also be utilized in cases of severe or refractory sicca and joint disease. Severe or refractory systemic disease with signs of end-organ damage due to pSD may also be considered for B-cell depleting therapies such as rituximab [24,26].

## 4. Kidney Manifestations in Primary Sjogren Disease

The reported incidence of kidney involvement in patients with pSD varies and ranges from 1 to 33% with approximately 14–25% of all pSD patients experiencing nephrolithiasis [27,28]. Kidney involvement in pSD results from two distinct pathophysiologic processes: epithelial disease with predominantly mononuclear lymphocytic infiltration resulting in tubulointerstitial nephritis (TIN) and non-epithelial disease with a secondary immune complex-mediated process resulting in glomerulopathy [29]. Tubulointerstitial nephritis (TIN) is the most common renal complication in pSD, and chronic kidney disease (CKD) due to TIN is the most common presentation [30,31]. TIN can further cause various defects in tubular function, including distal renal tubular acidosis (dRTA), nephrogenic diabetes insipidus, and rarely, proximal tubular abnormalities and Fanconi’s syndrome [32]. A few case reports of acquired Gitelman and Bartter syndromes associated with pSD have also been reported [29]. Glomerular disease in SD is less common. When present, the most common pattern of injury is membranoproliferative glomerulonephritis, typically caused by the production of a monoclonal IgM kappa paraprotein with rheumatoid factor activity that leads to type II mixed cryoglobulinemia [33]. Patients typically present with hematuria, proteinuria, hypertension, and various degrees of kidney impairment. An overview of these renal manifestations in pSD can be reviewed in Table 3.

## 5. Distal Renal Tubular Acidosis and Nephrolithiasis Review

Up to 25 percent of patients with SD have some defect in distal acidification, and SD is also one of the most common causes of acquired distal renal tubular acidosis (dRTA) [32,34]. Distal renal tubular acidosis (dRTA) occurring within the distal nephron is also known as Type I RTA as it was the first form of RTA described, and dRTA with subsequent kidney stone production remains a difficult-to-treat entity in patients with pSD [35].

Typically, acid (proton) secretion takes place in type A (alpha) intercalated cells when protons (H+) are produced in the cytosol by converting H_2_O and CO_2_ into protons and bicarbonate [35]. Thereafter, H+ is excreted and retained in the lumen of the collecting duct by binding with ammonia (NH_3_) to form ammonium (NH_4_), which can be excreted in the urine [35]. Consequently, dRTA manifests as a failure to properly excrete protons from type A intercalated cells in the connecting tubule and collecting duct, resulting in diminished urinary acidification (incapacity to generate urine pH below 5.5) and ammonium excretion [35]. dRTA may either be complete, leading to systemic metabolic acidosis and excessively alkaline urine, or incomplete, where the acidification defect is insufficient to cause overt acidosis [29]. In patients with dRTA, lab results typically demonstrate a normal anion gap metabolic acidosis with hypokalemia (for which the mechanism remains unknown) [36].

dRTA is also commonly associated with hypercalciuria, nephrocalcinosis, and/or nephrolithiasis [29]. dRTA promotes the formation of calcium-containing kidney stones first through metabolic acidosis increasing urinary calcium levels. This process stems from increased mobilization of calcium from bone and inhibition of calcium transport mechanisms within the renal tubule [37]. Of note, acute acidosis leads to rapid dissolution of calcium carbonate and hydroxyapatite from bone, and chronic acidosis (acidosis > 24 h) stimulates osteoclasts and inhibits osteoblasts, resulting in calcium release into the bloodstream [37]. Additionally, metabolic acidosis induces renal calcium wasting by directly affecting calcium handling within the renal tubule as it inhibits tubular calcium reabsorption, independent of parathyroid hormone and vitamin D [37]. Thus, these driving mechanisms precipitate increased urinary calcium. While urinary calcium levels climb, dRTA also causes a decrease in protective urinary citrate (due to increased citrate reabsorption from the proximal tubule mediated by acidosis), and an alkaline urine pH that facilitates calcium-phosphate precipitation and, thus, the development of calcium-phosphate-containing kidney stones (apatite stones) [38,39]. These co-occurring mechanisms thereby facilitate prominent nephrolithiasis in the setting of dRTA.

## 6. Evaluation and Management of Kidney Disease and Nephrolithiasis in Primary Sjogren Disease

In patients who experience pSD with systemic involvement but no identifiable renal disease, annual screening tests including serum creatinine, serum electrolytes, and urinalysis are advised for monitoring of kidney function [28]. If abnormalities are detected and kidney disease is present, more frequent monitoring (every 6 months) or imaging (such as renal ultrasound) should be considered to characterize and track dysfunction. The subsequent evaluation and management of kidney disease depends on the clinical presentation. Tubulointerstitial nephritis (TIN) should be suspected in patients presenting with sub-nephrotic levels of proteinuria or chronic kidney disease [28]. A kidney biopsy is usually required to confirm the diagnosis, guide immunosuppressive therapy, and assist with prognostication [28,40]. Of note, focal or diffuse mononuclear cell interstitial infiltration with varying degrees of tubular atrophy and fibrosis is typically seen on pathology in patients with SD-mediated TIN; however, this pattern is not specific only to SD [40]. Additionally, patients who instead present with features of glomerulonephritis also merit biopsy for thorough investigation into the underlying disease process. Testing for cryoglobulins and viral hepatitis is also recommended in this setting [28]. Lastly, while there is increasing interest in elucidating the role of innovative biomarkers such as neutrophil gelatinase-associated lipocalin (NGal) and non-coding RNA (ncRNA) in the pathogenesis and progression of renal disease in general, their role in SD-related renal disease has yet to be well clarified.

For patients with dRTA, nephrolithiasis, nephrocalcinosis, or isolated electrolyte disturbances with overall preserved eGFR the role for kidney biopsy remains unclear. In such cases, kidney biopsy may be deferred while closely monitoring kidney function and can later be pursued if pathology becomes more clinically significant [28]. Stone analysis, however, should be performed at least once to characterize the composition of associated kidney stones should they arise [41]. A bone mineral density scan should also be considered when providing holistic care, given the deleterious effects of chronic metabolic acidosis on bone mineralization [28].

Treatment of TIN due to pSD typically involves immunosuppressive therapy with glucocorticoids being the first choice [42]. Evidence for dosing or duration remains limited, with most reports suggesting the use of prednisone up to 1mg/kg per day for four to six weeks as initial therapy [42]. Azathioprine, mycophenolate mofetil, cyclophosphamide, and rituximab have been used as second-line or steroid-sparing agents [28,42]. Additionally, both glucocorticoids and second-line agents have been used in the treatment of glomerulonephritis in SD [28].

The goal of therapy in patients with nephrolithiasis and nephrocalcinosis is to reduce urinary calcium and phosphate excretion and increase urinary citrate excretion. This is usually achieved with alkali therapy for the correction of acidemia. Potassium citrate or potassium bicarbonate therapy is preferred, as most patients also have hypokalemia. Alkali therapy should be titrated to achieve low to normal serum bicarbonate levels without a substantial increase in urinary pH, which could lead to the precipitation of calcium phosphate [28]. Typical doses range from 20 to 80 milliequivalents per day, usually titrated based on serum bicarbonate and urine pH levels. Thiazide diuretics may also be used to decrease urinary calcium excretion [43]. General measures for stone prevention and recurrence, including increased fluid intake, reduced salt intake, and other dietary measures, should be recommended for all patients with kidney stones [43]. Interestingly however, the use of immunosuppressive therapy has not been thoroughly studied for pSD-related dRTA, nephrolithiasis, or nephrocalcinosis in the absence of TIN and other systemic indications.

## 7. Conclusions

In sum, Primary Sjogren Disease can manifest with both classic exocrine and cryptic systemic manifestations. Highly characteristic sicca symptoms and arthritis paired with positive serologies heavily aid in diagnosis, and it is important to recognize systemic disease should it arise. Furthermore, appropriate workup of renal dysfunction both in patients with and without pSD can aid in determining the underlying etiology for kidney disease. Immunosuppressive medication remains a hallmark of treatment in pSD-mediated TIN and glomerulonephritis, but isolated dRTA and nephrolithiasis in patients with pSD remain difficult to treat, and standard of care employs basic management principles. Additionally, hydroxychloroquine remains a first-line agent for treatment of classic sicca and arthritis symptoms in pSD. Overall, the mechanisms by which dRTA and nephrolithiasis arise are complex, and further research into management and pathophysiology, particularly in patients with pSD, is needed.

## Figures and Tables

**Table 1:** Case review data.

Author, Patient	Sex	Age at pSD Diagnosis	Age at Nephrolithiasis	Positive Serology	CKD	Evidence of RTA	Treatment
Schroeder, 1	M	41	46	SSA, SSB, RF	No	No	HCQ
Schroeder, 2	F	12	8	ANA, SSA, RF	No	No	None
Schroeder, 3	F	Unknown	Unknown	ANA, CCP	Yes	No	HCQ, LEF, Anti-TNFa
Schroeder, 4	F	17	20	SSA, SSB	No	Unknown	HCQ
Schroeder, 5	F	30	Unknown	ANA, SSA, SSB	Yes	Yes	HCQ, cevimeline
Schroeder, 6	F	38	Unknown	ANA	No	Yes	HCQ
Schroeder, 7	F	22	24	ANA, SSA, SSB, RF	Yes	Yes	HCQ
Baenas, 8	F	32	62	ANA, SSA, SSB	No	Yes	HCQ, MTX, prednisone
Morishita, 9	M	25	25	ANA, SSA, SSB	Yes	Yes	Unknown
Martin, 10	F	39	39	ANA, SSA, SSB	No	Yes	HCTZ
Aguilera, 11	F	32	27	ANA, SSA, RF	No	Yes	HCQ
Aguilera, 12	F	35	18	ANA	No	Yes	HCQ
Aguilera, 13	F	35	30	ANA, SSA, RF	No	Yes	HCQ

ANA: Antinuclear Antibody; SSA: Anti-Ro; SSB: Anti-La; RF: Rheumatoid Factor; CCP: Anti-Citric Citrullinated Peptide; HCQ: Hydroxychloroquine; LEF: Leflunomide; MTX: Methotrexate; and HCTZ: Hydrochlorothiazide.

**Table 2:** pSD Diagnostic and classification criteria.

2002 AECG Criteria [13]	2016 ACR/EULAR Criteria [14]	
		Score
I. Ocular Symptoms (at least one) - Symptoms of dry eyes for at least 3 months - A foreign body sensation in the eyes - Use of artificial tears 3 or more times per day II. Oral Symptoms (at least one) - Symptoms of dry mouth for at least 3 months - Recurrent or persistently swollen salivary glands - Need for liquids to swallow dry foods III. Ocular Signs (at least one) - Abnormal Schirmer’s test (without anesthesia; ≤5 mm/5 min) - Positive vital dye staining of the eye surface IV. Histopathology - Lip biopsy showing focal lymphocytic sialadenitis (focus score ≥1 per 4 mm^2^) V. Oral Signs (at least one) - Unstimulated whole salivary flow (≤1.5 mL in 15 min) - Abnormal parotid sialography - Abnormal salivary scintigraphy VI. Autoantibodies (at least one) - Anti-SSA (Ro) or Anti-SSB (La), or both	Labial salivary gland with focal lymphocytic sialadenitis and focus score of ≥1 foci/4 mm^2^	3
	2. Positive Anti-Ro/SSA	3
	3. Ocular Staining Score ≥5 (or van Bijsterveld score ≥4) in at least one eye	1
	4. Schirmer’s test ≤5 mm/5 min in at least one eye	1
	5. Unstimulated whole saliva flow rate ≤0.1 mL/min	1
	For inclusion in ACR/EULAR criteria, patients must have at least one symptom of ocular or oral dryness defined as the following: - Daily, persistent/symptomatic dry eyes for greater than 3 months - Recurrent sensation of sand/gravel in the eye - Use tear substitutes > than three times a day - Daily dry mouth for more than 3 months - Frequently drink liquids to aid in swallowing dry food	
Diagnosis for Primary Sjogren Disease: - Any 4 of the 6 items, and must include either item IV (Histopathology) or VI (Autoantibodies)	Diagnosis for Primary Sjogren Disease: - A total score of 4 or higher	
Exclusion Criteria: Past head and neck radiation treatment/Hepatitis C infection/Acquired immunodeficiency syndrome (AIDS)/Pre-existing lymphoma/Sarcoidosis/Graft versus host disease/Current use of anticholinergic drugs	Exclusion Criteria: History of head-and-neck radiation treatment/Active hepatitis C infection (with confirmation by polymerase chain reaction [PCR]) testing/AIDS/Sarcoidosis/Amyloidosis/Graft versus host disease/IgG4-related disease	

**Table 3:** Renal manifestations of pSD.

Mechanism	Renal Complications	Clinical Presentation
Epithelial Tubular disease Mononuclear lymphocytic infiltration leading to Tubulointerstitial nephritis (TIN)	Progressive Chronic Kidney Disease (CKD)	Urinalysis is nonspecific, can show sterile pyuria, mild proteinuria, or be unremarkable
	Distal Renal Tubular Acidosis (dRTA)	Metabolic acidosis, hypokalemia, osteomalacia, growth failure, nephrolithiasis, nephrocalcinosis
	Proximal Tubular Dysfunction/Fanconi Syndrome	Metabolic acidosis, hypokalemia, tubular proteinuria, aminoaciduria, glycosuria, phosphaturia, osteomalacia, growth failure
	Nephrogenic Diabetes Insipidus/Concentrating Defects	Asymptomatic, polyuria, nocturia
	Other rare tubular defects such as acquired Bartter or Gitelman-like syndrome are also possible	
Non-epithelial Glomerular disease Immune complex and antibody-mediated glomerular disease and vasculitis	Cryoglobulinemia-related Membranoproliferative Glomerulonephritis (MPGN)	Nephritic syndrome, hematuria, proteinuria, hypertension, and varying degrees of kidney function impairment
	Other rarer forms of glomerulonephritis such as IgA nephropathy and ANCA-associated pauci-immune glomerulonephritis are also possible	

## Data Availability

The original contributions presented in this study are included in the article. Further inquiries can be directed to the corresponding author.
